# Dimerization of the Vacuolar Receptors AtRMR1 and -2 from *Arabidopsis thaliana* Contributes to Their Localization in the *trans*-Golgi Network

**DOI:** 10.3390/ijms17101661

**Published:** 2016-09-30

**Authors:** Alessandro Occhialini, Guillaume Gouzerh, Gian-Pietro Di Sansebastiano, Jean-Marc Neuhaus

**Affiliations:** 1 Plant Biology and Crop Science, Rothamsted Research, Harpenden, AL5 2JQ Herts, UK; 2 Laboratory of Cell and Molecular Biology, Institute of Biology, University of Neuchâtel, Rue Emile-Argand 11, CH-2009 Neuchâtel, Switzerland; guillaume.gouzerh@unine.ch; 3 DISTEBA, Department of Biological and Environmental Sciences and Technologies, University of Salento, Campus Ecotekne, 73100 Lecce, Italy; gp.disansebastiano@unisalento.it

**Keywords:** AtRMR, PA domain, RING-H2, Ser-Rich domain, linker, transmembrane, plant secretory pathway, *trans*-Golgi network, endoplasmic reticulum, dimerization, laser scanning confocal microscopy, Bimolecular Fluorescent Complementation, *Arabidopsis thaliana*, *Nicotiana benthamiana*

## Abstract

In *Arabidopsis thaliana*, different types of vacuolar receptors were discovered. The AtVSR (Vacuolar Sorting Receptor) receptors are well known to be involved in the traffic to lytic vacuole (LV), while few evidences demonstrate the involvement of the receptors from AtRMR family (Receptor Membrane RING-H2) in the traffic to the protein storage vacuole (PSV). In this study we focused on the localization of two members of AtRMR family, AtRMR1 and -2, and on the possible interaction between these two receptors in the plant secretory pathway. Our experiments with agroinfiltrated *Nicotiana benthamiana* leaves demonstrated that AtRMR1 was localized in the endoplasmic reticulum (ER), while AtRMR2 was targeted to the *trans*-Golgi network (TGN) due to the presence of a cytosolic 23-amino acid sequence linker. The fusion of this linker to an equivalent position in AtRMR1 targeted this receptor to the TGN, instead of the ER. By using a Bimolecular Fluorescent Complementation (BiFC) technique and experiments of co-localization, we demonstrated that AtRMR2 can make homodimers, and can also interact with AtRMR1 forming heterodimers that locate to the TGN. Such interaction studies strongly suggest that the transmembrane domain and the few amino acids surrounding it, including the sequence linker, are essential for dimerization. These results suggest a new model of AtRMR trafficking and dimerization in the plant secretory pathway.

## 1. Introduction

Vacuolar proteins are targeted to the different types of vacuoles via organelles that communicate with each other by a highly regulated process involving different kinds of vesicles as well as cisternal maturation [1,2]. This transport mechanism is known as secretory pathway and comprises the endoplasmic reticulum (ER), the Golgi apparatus, the *trans*-Golgi network (TGN) and prevacuoles [1,3,4]. Soluble secretory proteins are synthesized with an N-terminal signal peptide (SP) that allows them to enter co-translationally the lumen of the ER [5], the first compartment of the plant secretory pathway, where folding and assembly of newly synthesized proteins take place [6,7]. After accumulation in this early compartment, secretory proteins can be transported through the Golgi apparatus to the *trans*-Golgi network (TGN) and to prevacuoles, which constitute important traffic points for proteins equipped with specific sequence elements known as vacuolar sorting determinants (VSDs) [4,8,9].

In the last few years, it has been demonstrated that many vacuolar proteins are sorted to vacuoles by cargo receptors that are transmembrane proteins localized to the TGN/prevacuolar system [10,11]. Such receptors are able to bind specific VSDs of vacuolar protein precursors, diverting them from the default pathway of secretion outside the plasma membrane and therefore, allowing their transport to the final destination [4,12,13]. Different types of vacuoles, including the lytic vacuole (LV) and the protein storage vacuole (PSV), have been shown to be the final destinations of vacuolar proteins [14,15,16,17]. It has been proposed that two different families of membrane receptors are involved in protein sorting to these vacuoles. The first family, the Vacuolar Sorting Receptors (VSRs) [13], is encoded by seven genes in *A. thaliana* (from *AtVSR1* to *AtVSR7*), while the second family, the Receptor Membrane RING-H2 (RMRs) [18], is encoded by six genes (from *AtRMR1* to *AtRMR6*). The RMRs are type I transmembrane proteins with a single transmembrane domain, that were discovered because they share an analogous PA domain (Protease-Associated Domain) with the VSRs. Their N-terminal luminal part consists mainly of the PA domain, which in VSRs is involved in binding the vacuolar proteins [18,19], whereas their C-terminal cytosolic part contains a particular RING domain, a RING-H2, of unknown function [18]. In other proteins, the RING-H2 domain is thought to be involved in protein-protein interactions and dimerization [20]. The RMR family of Angiosperms can be subdivided into two subfamilies (Supplementary Material Figure S1 and Table S1) represented in *A. thaliana* by AtRMR1 (At5g66160) and AtRMR2–6 (from AtRMR2 to AtRMR6), respectively. In many RMRs of the second subfamily (e.g., AtRMR2, -3, and -4, but not -5 and -6), and indeed in non-angiosperm plants, the RING-H2 domain is followed by a large Ser-Rich domain that contains several potential phosphorylation sites [21]. For instance, the 168 amino acids long Ser-Rich domain of AtRMR2 contains 51 serines, of which 43 have been identified as potential phosphorylation sites (NetPhos 3.1 Server, CBS, Technical University of Denmark, Lyngby, Denmark). On the contrary, the C-terminal luminal part of AtRMR1 contain a much shorter Ser-Rich tail of only 31 amino acids in which only 5 serines have been predicted to be potential phosphorylation sites (Supplementary Material Figure S2, for comparing the Ser-Rich domains of AtRMR1 and -2).

The VSR family is involved in vacuolar sorting to LV through interactions of its PA and VSR-specific domains with sequence specific VSDs (ssVSDs) located in the amino acid sequence of particular vacuolar proteins [12,22,23]. Less is known about the vacuolar receptors involved in protein targeting to the PSV. An important group of candidate receptors involved in such latter pathway of vacuolar sorting was proposed to be from the aforementioned RMR family [21,24,25]. In vitro interaction experiments between RMR and vacuolar protein that accumulate in the PSV (e.g., barley lectin, bean phaseolin and tobacco chitinase), together with the subcellular localization of these receptors, support this hypothesis [24,25]. In fact, it has been shown in *A. thaliana* that AtRMR1 is localized in prevacuolar compartments [24] or in vacuoles [26], while AtRMR2 (At1g71980), has been localized by immunogold electron microscopy in the late Golgi apparatus, dense vesicles (DV) and PSV in *A. thaliana* embryos [11].

Whether RMRs really are the receptors for protein sorting to PSV is still not clear and many questions about their trafficking and functions are still open. In this study we observed that in *N. benthamiana* leaves transformed by agroinfiltration, AtRMR2 was localized to the TGN, whereas AtRMR1 was mainly accumulated in the ER. By deleting and/or replacing different domains, we demonstrated that a cytosolic 23-amino acid sequence linker, located immediately after the transmembrane domain of AtRMR2, could act as a localization signal for the TGN. In fact, replacing the corresponding linker of AtRMR1 with the linker of AtRMR2, the chimaeric RMR was localized in AtRMR2-positive punctate structures, i.e., in the TGN. Furthermore, we demonstrated by Bimolecular Fluorescent Complementation (BiFC) that AtRMR2 forms homodimers and can also interact with AtRMR1 to form heterodimers, which then also localizes to the TGN. These experiments performed using AtRMR deletion mutants of different cytosolic (PA) and luminal (RING-H2 and Ser-Rich) domains, suggest that only the transmembrane domain and the neighboring sequences, including the sequence linker, are necessary for this dimerization.

## 2. Results

### 2.1. AtRMR1 and AtRMR2 Have Different Subcellular Localizations in N. benthamiana Leaves

**Figure 1:**
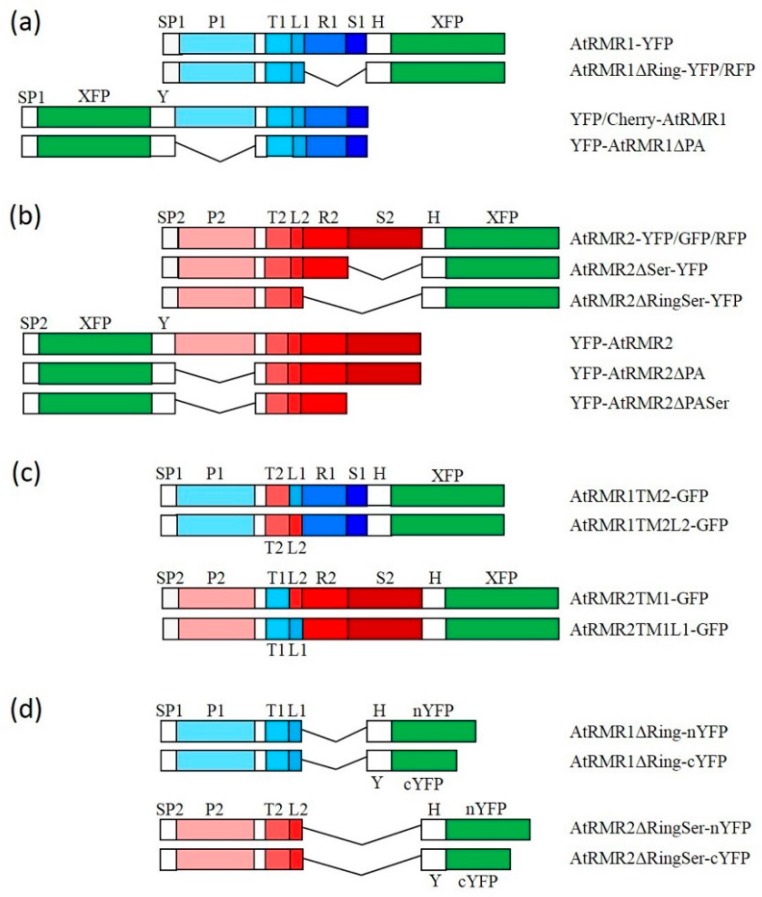
Schematic representation of the fluorescent AtRMR (Receptor Membrane RING-H2) fusion proteins generated in this work. All these fusion proteins were over-expressed in *Nicotiana benthamiana* leaves under the control of the cauliflower mosaic virus (CaMV) 35S promoter and terminator. (**a**) C- and N-terminal fusions of fluorescent proteins to AtRMR1 and to its mutants deleted for the Protease-Associated (PA) domain or the RING-H2 domain along with the short Ser-Rich tail; (**b**) C- and N-terminal fusions of fluorescent proteins to AtRMR2 and to its mutants deleted for the PA, the Ser-Rich or the RING-H2 domains; (**c**) AtRMR1 and -2 mutants with the transmembrane domain or both the transmembrane domain and the linker sequence swapped; and (**d**) constructions for Bimolecular Fluorescent Complementation (BiFC) detection of AtRMR1 and -2 lacking their cytosolic domains (RING-H2/Ser-Rich) and fused to either half of YFP. Abbreviations (numbered 1 or 2 for domains of each AtRMR): SP, signal peptide; P, PA-domain; T, transmembrane domain; L, linker sequence; R, RING-H2 domain; S, Ser-Rich domain; XFP, fluorescent protein (YFP, yellow fluorescent protein; RFP, red fluorescent protein; GFP, green fluorescent protein; or Cherry, mCherry fluorescent protein, as indicated); nYFP, N-terminal half of YFP; cYFP, C-terminal half of YFP; H, Gly_6_-HA epitope spacer; Y, Gly_6_-Myc epitope spacer. The main protein domains (P, T, L, R and S) of AtRMR1 and -2 are depicted with different shade of blue and red, respectively, while XFP is depicted in green.

**Figure 2:**
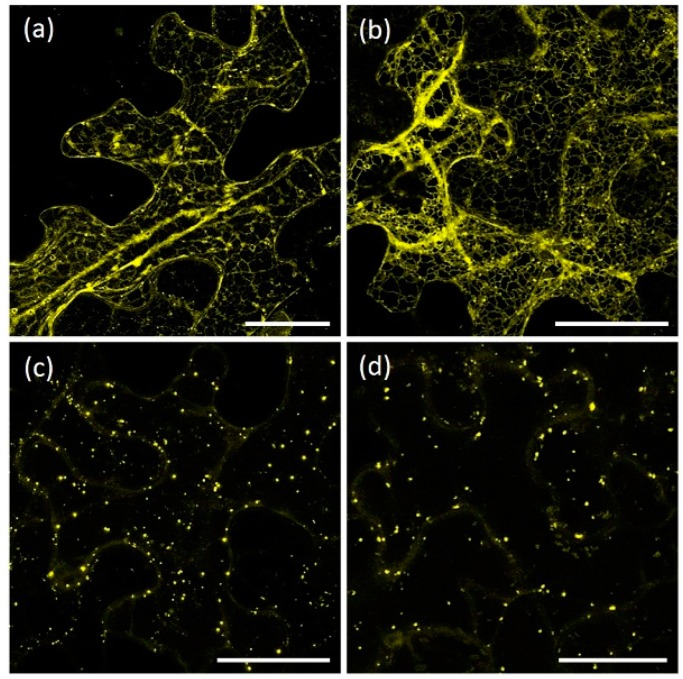
Localization of AtRMR1 and -2 in *N. benthamiana* leaves: (**a**,**b**) stacked confocal images of epidermal cells expressing either AtRMR1-YFP (**a**) or YFP-AtRMR1 (**b**); and (**c**,**d**) stacked confocal images of epidermal cells expressing either AtRMR2-YFP (**c**) or YFP-AtRMR2 (**d**). YFP signal (**a**–**d**). Scale bars = 30 µm.

For the localization of AtRMRs, the full-length AtRMR1 and AtRMR2 fused to the yellow fluorescent protein (YFP) at either C- or N-terminal ends (Figure 1a,b) were transiently expressed in *N. benthamiana* leaves under control of the 35S promoter. Unexpectedly, the localization patterns of the two related proteins were different and dissimilar from what previously reported [11,24]. In leaves expressing AtRMR1-YFP or YFP-AtRMR1, the localization pattern of the fluorescent images revealed a network structure typical of ER localization (Figure 2a,b), while in leaves expressing AtRMR2-YFP or YFP-AtRMR2, we observed a punctate pattern (Figure 2c,d).

**Figure 3:**
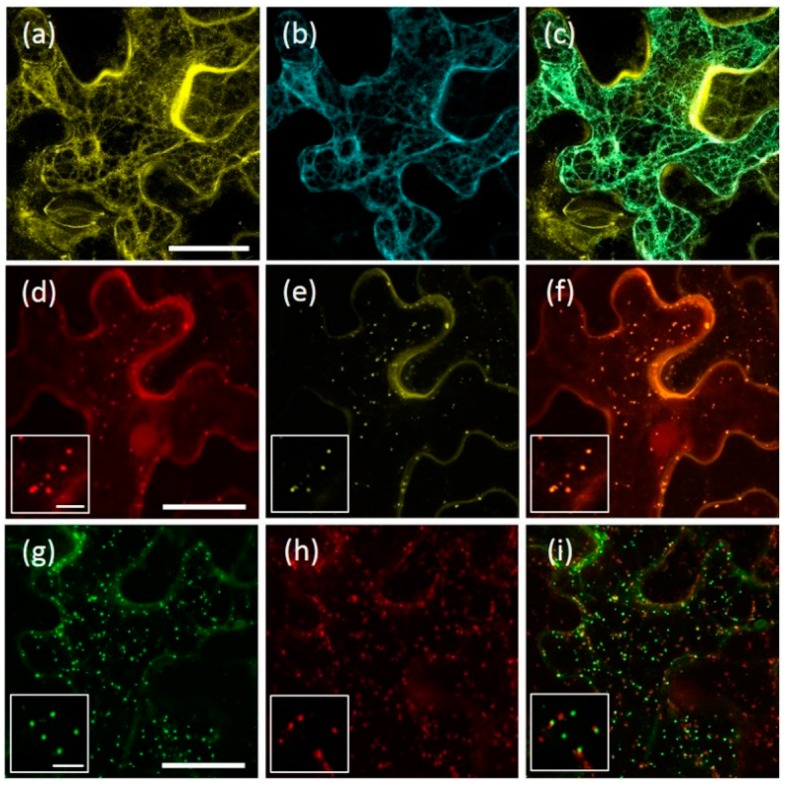
Localization of AtRMR1 and -2 in the endoplasmic reticulum (ER) and *trans*-Golgi network (TGN), respectively. Co-localization of AtRMR1-YFP with the ER marker p6-CFP (**a**–**c**); and co-localization of AtRMR2-RFP with the TGN marker Venus-SYP61 (**d**–**f**) or AtRMR2-GFP with the Golgi marker GONST1-RFP (**g**–**i**). YFP signal (**a**); CFP signal (**b**); RFP signal (**d**,**h**); Venus signal (**e**); GFP signal (**g**); and merged images (**c**,**f**,**i**). Stacked confocal images, excepted inserts (**d**–**i**; single images). Scale bars = 30 μm; and 5 μm (inserts).

The ER localization of the AtRMR1 fusion proteins was confirmed by full co-localization with the ER marker, p6-CFP (CFP, cyan fluorescent protein) [27] (Figure 3a–c). In contrast, in cells expressing AtRMR2-YFP and YFP-AtRMR2, the fluorescent punctate structures fully co-localized with the *trans*-Golgi network (TGN) marker SYP61 [28] (Figure 3d–f). Furthermore, AtRMR2 did not co-localize with the Golgi marker GONST1 but was often closely associated with it (Figure 3g–i) in agreement with the model that the Golgi apparatus dissociates from the TGN [28,29]. These experiments clearly demonstrate that two AtRMRs from different subfamilies localized to different subcellular compartments.

### 2.2. The Protease-Associated (PA), RING-H2 and Ser-Rich Domains Are Not Involved in the Subcellular Localization of Either AtRMR1 or AtRMR2

**Figure 4:**
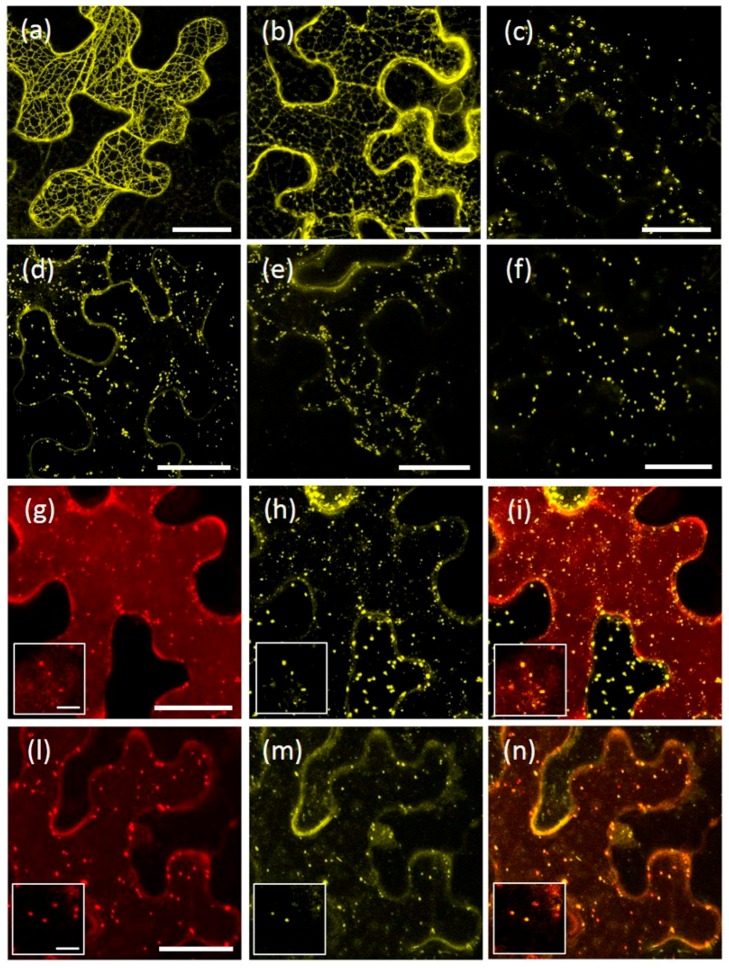
Unchanged localization of AtRMR1 and -2 deletion mutants. Stacked confocal images of epidermal cells expressing the fusion protein: AtRMR1ΔRing-YFP (**a**); YFP-AtRMR1ΔPA (**b**); AtRMR2ΔSer-YFP (**c**); AtRMR2ΔRingSer-YFP (**d**); YFP-AtRMR2ΔPA (**e**); and YFP-AtRMR2ΔPASer (**f**). Co-localization of AtRMR2 and its deletion mutants in *N. benthamiana* leaves (**g**–**n**): Stacked confocal images, excepted inserts (**g**–**n**; single images) of epidermal cells expressing AtRMR2-RFP and the deletion mutant AtRMR2ΔRingSer-YFP (**g**–**i**); or AtRMR2-RFP and the deletion mutant YFP-AtRMR2ΔPASer (**l**–**n**). RFP signal (**g**,**l**); YFP signal (**a**–**f,h,m**); and merged images (**i**,**n**). Scale bars = 30 µm; and 5 μm (inserts).

To characterize which domains of AtRMRs are involved in protein localization, a number of deletion mutants of the PA, RING-H2 and Ser-Rich domains were generated. For AtRMR1, a deletion mutant lacking the RING-H2 domain as well as the short Ser-Rich tail, fused at the C-terminus with YFP (AtRMR1ΔRing-YFP), and a second one lacking the PA domain, fused at the N-terminus with YFP (YFP-AtRMR1ΔPA) were generated (Figure 1a). The fluorescence pattern of both showed that the modified forms of AtRMR1 were predominantly located in networks that occupied the cortical and inner part of the cell, typical of ER localization (Figure 4a,b). The ER pattern of the full-length protein was indistinguishable from the pattern of the two deletion mutants, demonstrating that neither PA nor RING-H2 domains along with the short Ser-Rich tail were involved in AtRMR1 localization.

A similar approach was also used to characterize the protein domains of AtRMR2. For this purpose, the following mutants were generated (Figure 1b): two AtRMR2 mutants fused with YFP at the C-terminal end and lacking either the Ser-Rich domain (AtRMR2ΔSer-YFP) or both RING-H2 and Ser-Rich domains (AtRMR2ΔRingSer-YFP) respectively, together with two mutants fused to YFP at the N-terminus and lacking either the PA domain (YFP-AtRMR2ΔPA) or the PA and the Ser-rich domains (YFP-AtRMR2ΔPASer). All these fusion proteins were localized to punctate structures similar to those observed for the full-length AtRMR2 (Figure 4c–f). Two different co-localization experiments between AtRMR2-RFP and either AtRMR2ΔRingSer-YFP (Figure 4g–i) or YFP-AtRMR2ΔPASer (Figure 4l–n), demonstrated that both mutants mainly co-localized with the full-length AtRMR2 in the TGN. These results demonstrated that neither the PA, nor the RING-H2, nor the Ser-Rich domains of AtRMR2 were essential for protein localization.

### 2.3. The Transmembrane Domain Is Not Involved in AtRMRs Localization

**Figure 5:**
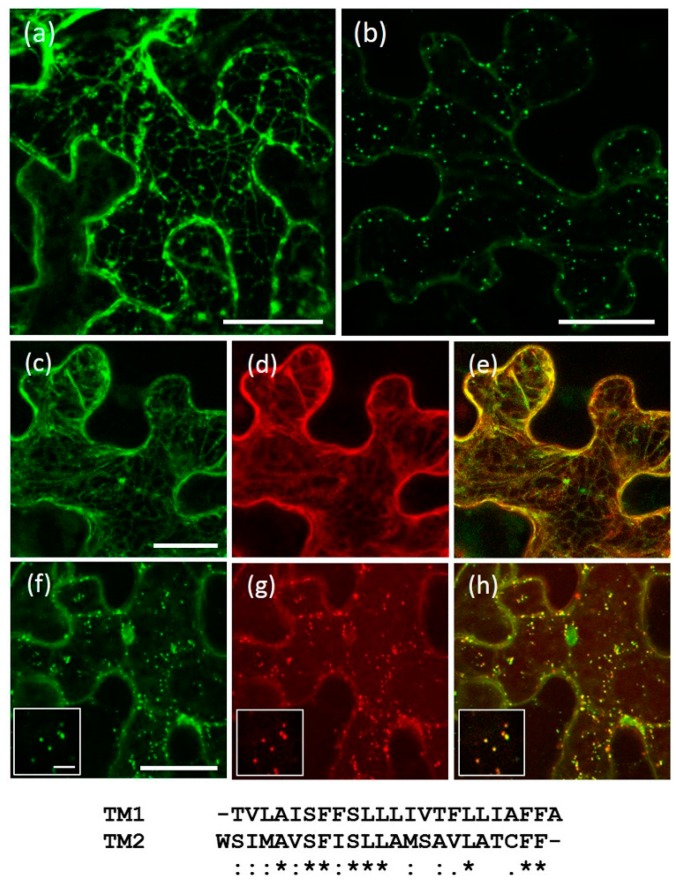
Localization of AtRMR1 and -2 with swapped transmembrane domains. Stacked confocal images of epidermal cells expressing AtRMR1 with the transmembrane domain of AtRMR2 (**a**; AtRMR1TM2-GFP) or AtRMR2 with the transmembrane domain of AtRMR1 (**b**; AtRMR2TM1-GFP). Stacked confocal images, excepted inserts (**f**–**h**; single images), of epidermal cells co-expressing AtRMR1TM2-GFP with Cherry-AtRMR1 (**c**–**e**) or AtRMR2TM1-GFP with AtRMR2-RFP (**f**–**h**). GFP signal (**a**–**c**,**f**); RFP signal (**d**,**g**); and merged images (**e**,**h**). Scale bars = 30 μm; and 5 μm (inserts). An alignment of the transmembrane sequences TM1 and TM2 is shown below the figure. (*) Identical amino acids; (:) amino acids with similar biochemical properties; (.) amino acids with semi-similar biochemical properties.

The 23 residue transmembrane domains (TM, as delimited by TMHMM Server v. 2.0, CBS, Technical University of Denmark, Lyngby, Denmark) of AtRMR1 and -2 were tested for the presence of putative localization signals by exchanging them in the full length AtRMR1 and -2 and observing the effects on their localization. For this purpose, two C-terminal fusion proteins with GFP (green fluorescent protein) were generated (Figure 1c): AtRMR1 with the TM of AtRMR2 (AtRMR1TM2-GFP), and AtRMR2 with the TM of AtRMR1 (AtRMR2TM1-GFP). For both fusion proteins we observed the same subcellular localization as the respective wild-type proteins, since the fluorescence was localized to the ER network (AtRMR1TM2-GFP) and to punctate structures (AtRMR2TM1-GFP) (Figure 5a,b). These unchanged localizations were confirmed by the perfect co-localization between these two mutants and their respective wild-type proteins (Figure 5c–h), suggesting that the transmembrane domain of AtRMRs was not involved in protein localization.

### 2.4. A Short Sequence Linker of AtRMR2 Is Involved in Protein Trafficking to the trans-Golgi Network (TGN)

**Figure 6:**
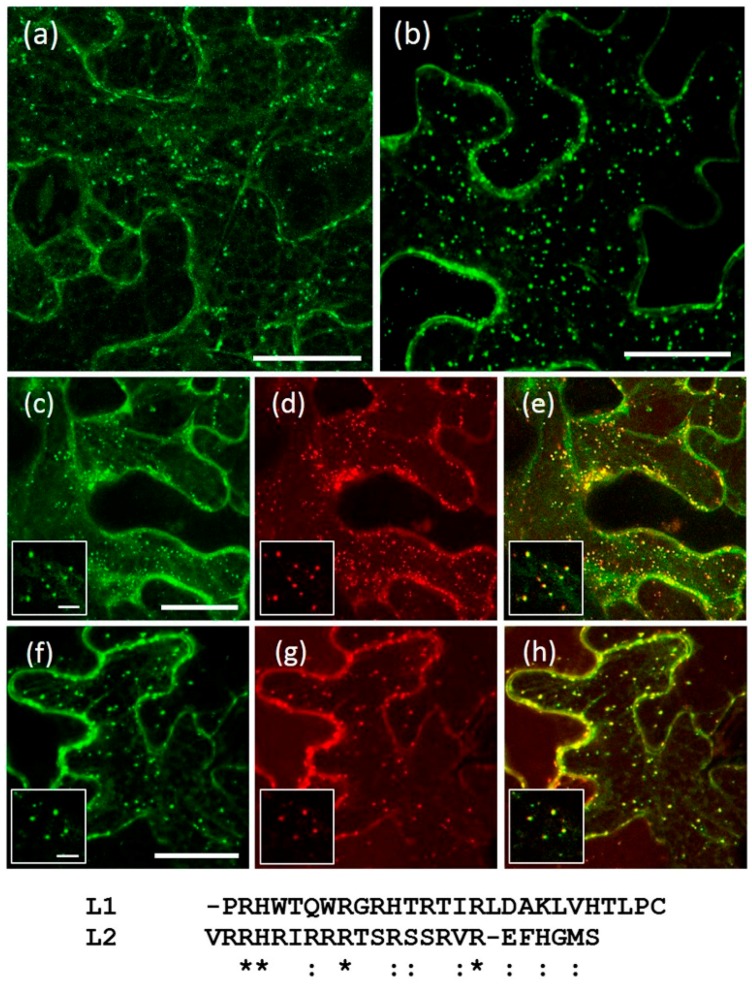
Localization of AtRMR1 and -2 with swapped transmembrane domains + linkers. Stacked confocal images of epidermal cells expressing AtRMR1 with the transmembrane and linker of AtRMR2 (**a**; AtRMR1TM2L2-GFP); or AtRMR2 with the transmembrane and linker of AtRMR1 (**b**; AtRMR2TM1L1-GFP). Stacked confocal images, excepted inserts (**c**–**h**; single images), of epidermal cells co-expressing AtRMR1TM2L2-GFP with AtRMR2-RFP (**c**–**e**) or co-expressing AtRMR2TM1L1-GFP with AtRMR2-RFP (**f**–**h**). GFP signal (**a**–**c**,**f**); RFP signal (**d**,**g**); and merged images (**e**,**h**). Scale bars = 30 μm; and 5 μm (inserts). An alignment of the linker sequences L1 and L2 is shown below the figure. (*) Identical amino acids; (:) amino acids with similar biochemical properties.

Having excluded the luminal PA domain, the transmembrane domain and the cytosolic RING-H2 and Ser-rich domains, the sought-after localization signals still could be within the short cytosolic linker (~23 residues) located between the TM and RING-H2 domains in the cytosolic part of AtRMRs. The functional characterization of the AtRMR1 and AtRMR2 linkers (L1 and L2, respectively) was performed by observing the localization effects caused by exchanging these two sequences in the full-length AtRMR1 and -2. Two more mutants were generated: AtRMR1TM2L2-GFP, in which the TM1 and L1 of AtRMR1 were replaced by the corresponding sequences from AtRMR2 (TM2 + L2), and the reverse mutant AtRMR2TM1L1-GFP, where the TM2 and L2 of AtRMR2 were replaced by TM1 + L1 (Figure 1c). The AtRMR1TM2L2 mutant was able to exit the ER, becoming localized within the same punctate structures as wild-type AtRMR2 (Figure 6a,c–e), indicating that the linker L2 contains a motif involved in trafficking to TGN. Unexpectedly, the reverse mutant AtRMR2TM1L1 still localized to the punctate structures (Figure 6b,f–h), suggesting that the localization signal located in L2 appears to be redundant with another signal of unidentified location within AtRMR2.

### 2.5. AtRMR1 Changes Localization When Co-Expressed with AtRMR2

By analogy with receptors, such as VSRs [30], it is possible that AtRMRs participate in oligomerization or interact with other proteins in the endomembrane system. To probe interactions between AtRMR1 and -2, we performed co-expression experiments using AtRMR proteins fused to different fluorescent reporters (AtRMR2-RFP and AtRMR1-YFP), and looked for overlapping or altered AtRMR localization as evidence for protein–protein interaction.

**Figure 7:**
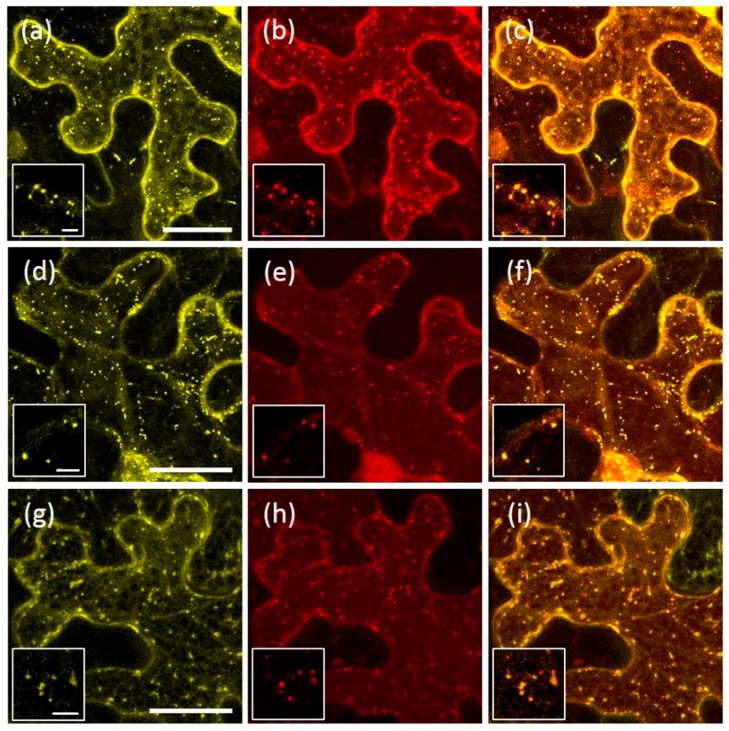
Co-expression of AtRMR2-RFP with either AtRMR1-YFP or its deletion mutants. Stacked confocal images, excepted inserts (**a**–**i**; single images), of epidermal cells expressing the following combinations: (**a**–**c**) AtRMR1-YFP and AtRMR2-RFP; (**d**–**f**) AtRMR1ΔRing-YFP and AtRMR2-RFP; and (**g**–**i**) YFP-AtRMR1ΔPA and AtRMR2-RFP. YFP signal (**a**,**d**,**g**); RFP signal (**b**,**e**,**h**); and merged images (**c**,**f**,**i**). Scale bars = 30 µm; and 5 μm (inserts).

In *N. benthamiana* cells expressing only AtRMR2-RFP or AtRMR1-YFP, each fusion protein showed the same localization as seen previously. In contrast, in cells expressing both fusion proteins, AtRMR1-YFP co-localized in punctate structures with AtRMR2-RFP, in addition to the normal ER labeling (Figure 7a–c). This result indicates an interaction between these different AtRMR receptors and that this leads to the export of AtRMR1-YFP/AtRMR2-RFP complexes from the ER to a post-Golgi compartment (TGN). We then investigated which domains of AtRMRs were involved in this protein–protein interaction and thus we co-expressed the full-length AtRMR2-RFP with AtRMR1ΔRing-YFP (lacking both the C-terminal RING-H2 domain and the short Ser-Rich tail, Figure 7d–f) or YFP-AtRMR1ΔPA (lacking the N-terminal PA domain, Figure 7g–i), respectively. In both co-localization experiments the two AtRMR1 mutants were re-localized to AtRMR2-RFP-labeled punctate structures, showing that neither the PA nor the RING-H2 and Ser-Rich domains were involved in protein–protein interaction, and that only the transmembrane domain and/or the neighboring sequences, including the linker, were involved in these interactions.

### 2.6. Homo- and Hetero-Dimerization of AtRMRs

**Figure 8:**
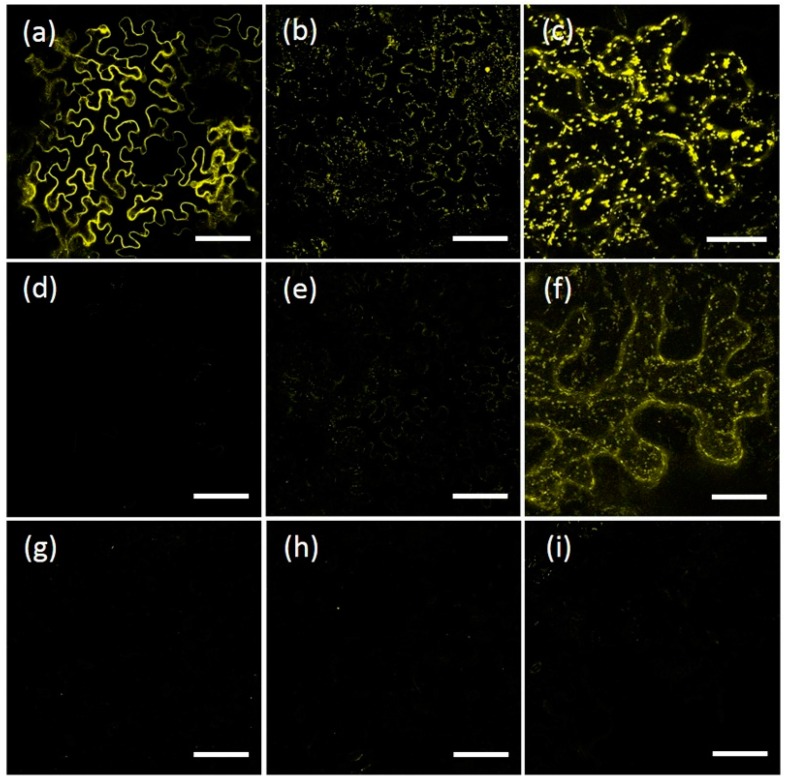
Dimerization of AtRMRs tested by BiFC. Confocal images of epidermal cells expressing split-YFP constructs: (**a**) The positive control, p6-nYFP and p6-cYFP; (**b**,**c**) AtRMR2ΔRingSer-nYFP and AtRMR2ΔRingSer-cYFP; (**d**) AtRMR1ΔRing-nYFP and AtRMR1ΔRing-cYFP; (**e**–**f**) AtRMR2ΔRingSer-nYFP and AtRMR1ΔRing-cYFP; (**g**) negative control, AtRMR2ΔRingSer-nYFP alone; (**h**) negative control, AtRMR2ΔRingSer-cYFP alone; and (**i**) negative control, AtRMR2ΔRingSer-nYFP and p6-cYFP. Singles images showing an overview of leaf tissue expressing the indicated fusion proteins (**a**,**b**,**d**,**e**,**g**–**i**), excepted stacked confocal images showing the localization in punctate structures of AtRMR2ΔRingSer homodimers and AtRMR2ΔRingSer-AtRMR1ΔRing heterodimers in a single epidermal cell, respectively (**c**,**f**). YFP signal (**a**–**i**). Scale bars = 100 μm (**a**,**b**,**d**,**e**,**g**–**i**); 30 µm (**c**,**f**).

A Bimolecular Fluorescence Complementation (BiFC) technique [31,32] was used to investigate AtRMR dimerization in *N. benthamiana* leaf cells. Since the previous results suggested that the PA, RING-H2 and Ser-Rich domains were not involved in protein–protein interactions, we performed BiFC using the two deletion mutants, AtRMR1ΔRing and AtRMR2ΔRingSer. Moreover, the constructs lacking the Ring-H2 domain are more expressed (or more stable) facilitating the detection of the split-YFP signal. These two mutants were then fused to two non-fluorescent N-terminal and C-terminal YFP fragments (nYFP and cYFP), generating two pairs of fusion proteins for BiFC (split AtRMR1ΔRing-nYFP/cYFP and split AtRMR2ΔRingSer-nYFP/cYFP, Figure 1d). For positive controls, we tested the ER-localized viral protein p6 (split p6-nYFP/cYFP; Figure 8a) while for negative controls we tested separately the single fusion proteins (AtRMR2ΔRingSer-nYFP in Figure 8g or AtRMR2ΔRingSer-cYFP in Figure 8h) or tested one of them (AtRMR2ΔRingSer-nYFP) with p6-cYFP (Figure 8i).

In leaves expressing AtRMR2ΔRingSer-nYFP and -cYFP, a strong BiFC signal was observed supporting homo-dimerization between AtRMR2 receptors (Figure 8b,c). On the contrary, a fluorescent signal higher than in the negative controls was not visualized in leaves expressing AtRMR1ΔRing-nYFP and -cYFP (Figure 8d), suggesting that AtRMR1 cannot make homo-dimers. However, a clear signal was also observed when co-expressing AtRMR2ΔRingSer and AtRMR1ΔRing fused to complementary YFP fragments (Figure 8e,f) supporting the ability of these two AtRMR receptors to produce heterodimers. Such experiments also support previous results of AtRMRs co-localization and the evidence that the TM and/or the amino acids surrounding it, including L, are the domains involved in AtRMR dimerization.

### 2.7. AtRMR2 Homodimers Do Not Interact with AtRMR1 to Form Trimers or Tetramers

**Figure 9:**
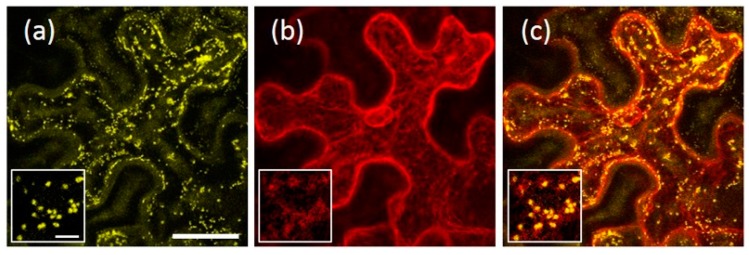
AtRMRs oligomerization. AtRMR trimer or tetramer formation tested by combined experiments of BiFC and co-expression in *Nicotiana benthamiana* leaves. Stacked confocal images, excepted inserts (**a**–**c**, single images), of epidermal cells expressing AtRMR2ΔRingSer-nYFP, AtRMR2ΔRingSer-cYFP and AtRMR1ΔRing-RFP. (**a**) YFP signal; (**b**) RFP signal; and (**c**) merged images. Scale bars = 30 µm; and 5 μm (inserts).

We performed combined experiments of BiFC and co-expression to investigate possible oligomerization (to trimers or tetramers) between two AtRMR2 and at least one AtRMR1. For this purpose, the two fusion proteins for BiFC, AtRMR2∆RingSer-nYFP and AtRMR2∆RingSer-cYFP, were co-expressed in the same *N. benthamiana* cells with AtRMR1∆Ring-RFP. In this case, a positive split-YFP signal was observed in punctate structures, while ER localization of AtRMR1∆Ring-RFP was observed (Figure 9). Such a fluorescent pattern may suggest that AtRMR2 preferentially homodimerizes (possibly further stabilized by the association of the two complementary YFP halves) and does not further associate with AtRMR1 to form trimers or tetramers, and therefore AtRMR1 cannot be transported to the TGN.

## 3. Discussion

### 3.1. AtRMRs Localize in Different Compartments of the Secretory Pathway

In agroinfiltrated *N. benthamiana* leaves both full-length AtRMR1 fusion proteins (AtRMR1-YFP and YFP-AtRMR1) localized to the cortical ER network that extended into the cell (Figure 2a,b). This localization was confirmed by the perfect overlap with the ER marker protein p6-CFP [27] (Figure 3a–c). By contrast, the full length AtRMR2 fused to YFP at either C- or N-terminus was localized in the TGN, as supported by the perfect co-localization with the TGN marker SYP61 [28] (Figure 2c,d and Figure 3d–f).

We also observed that the AtRMR2-YFP-labeled compartments did not co-localize with the Golgi marker GONST1 (Figure 3g–i), although in some cases they were found to be in close proximity. The Golgi apparatus and TGN have been demonstrated to be spatially separated organelles, but they are sometimes detected in close proximity [28,29].

Fusion of YFP at either ends of both AtRMR1 and -2 did not alter their localization, suggesting that these receptors do not possess N- or C-terminal localization sequences that could have been masked by the fluorescent reporter. While AtRMR2 localization in the TGN is partially compatible with previously described localization [11], for AtRMR1 the distribution observed in our experimental system is different to both the prevacuolar compartment (PVC) (and to a lesser extent Golgi) localization described by Park et al. [24] and the localization in vacuoles described by Scabone et al. [26]. Such differences in AtRMR1 localization could be due to the different experimental system and plant model (transient expression in *A. thaliana* protoplasts; see discussion below) used by Park et al. [24]. The results of Scabone et al. [26] could be due to the use of N-terminal (luminal) RFP fusion proteins. We have ourselves experienced that RFP tag position can affect the localization of a transmembrane protein. We experienced RFP effect during the expression of membrane-anchored HIV p24 (24 kDa-capsid-protein from human immunodeficiency virus) in plants; two versions of the luminal domain were tested. These experiments have shown that the fusion protein p24-RFP-TM localized to the expected compartment, while the reversed fusion protein RFP-p24-TM accumulated in the vacuole [33,34].

### 3.2. The Main Protein Domains of AtRMRs, Including the Transmembrane Domain, Are Not Involved in Protein Localization

AtRMR1 and AtRMR2 belong to different subfamilies of RMRs and have different subcellular localizations, suggesting the presence of different signals and mechanisms involved in their trafficking. The expression in *N. benthamiana* leaves of AtRMR1 and AtRMR2 deletion mutants lacking the PA, RING-H2 or Ser-Rich domains clearly showed that all these mutants had the same localization as the full-length wild-type proteins (Figure 4). These results demonstrated that the three main protein domains of AtRMRs, the PA, RING-H2 or Ser-Rich domains are not responsible of the subcellular localization of AtRMRs. This differentiate plant’s from animal homologs of RMRs, such as RNF13/167, which require their PA domain for their correct endosomal localization [35].

Although AtRMR1 and -2 have similar transmembrane domains (39% amino acid sequence identity), and the same predicted 23-residue length (alignment Figure 5), the two proteins have different localizations. The length of the TM alone can determine protein localization in plant cells [36] but by this criterion, AtRMR1 would have been expected to localize like AtRMR2 in a post-ER compartment. No change in protein localization was observed for either replacement mutant, AtRMR1TM2 or AtRMR2TM1, in which the TMs were swapped (Figure 5). This result showed that TM1 and TM2 did not define the localization of AtRMR2 and AtRMR1, respectively, and therefore, these sequences did not act as signals for the subcellular localization of these proteins. Furthermore, our results suggest that neither the length nor the amino acid sequence of AtRMR transmembrane domains were sufficient to determine protein localization. Therefore, other signals and/or mechanisms must determine the subcellular localization of these receptors.

### 3.3. The Sequence Linker of AtRMR2 Is Involved in the Protein Traffic to the TGN

Specific motifs involved in protein trafficking and recycling from the prevacuole back to the TGN have been already characterized in the cytosolic tail of the other family of vacuolar receptors, AtVSR (binding protein of 80 kDa (BP80) in *Pisum sativum*) [37,38]. Our results also suggested that a small sequence linker (L) of ~23 residues in the cytosolic part of AtRMR2, between the TM and the RING-H2 domains, is involved in protein localization. By exchanging the wild-type TM1 + L1 of AtRMR1 with the corresponding sequences from AtRMR2 (TM2 + L2), the resulting AtRMR1TM2L2 mutant was able to exit the ER, localizing to AtRMR2-positive punctate structures (Figure 6a,c–e). This result supports the presence of a localization signal in L2, sufficient to redirect an ER-resident protein to the TGN. Unexpectedly, when we exchanged the TM2 + L2 in AtRMR2 with TM1 + L1, the resulting AtRMR2TM1L1 mutant still accumulated in punctate structures (Figure 6b,f–h). This excludes an ER retention signal in AtRMR1 but suggests that the localization signal within L2 is redundant with another signal, which could be located in one of the previously deleted domains or in the short luminal linker between the PA and the TM domain, the only sequence that was not replaced or deleted in this study.

In the linker of AtRMR2 there is no typical Tyr or Ile-Met “dileucine” motif as found in the cytosolic tail of AtVSRs [37,38], but there are many arginines (R) (Figure 6). In fact, the first 17 residues of L2 contain nine arginines, compared to the five of L1. These arginines and particularly a pair and a triplet could constitute a dibasic motif. Such a motif has already been shown to be involved in ER export and Golgi localization of glycosyltransferases in animals [39] and plants [40]. Alternatively, the putative motif could include the three serine residues located in the AtRMR2 linker predicted to be phosphorylation sites (online software NetPhos 2.0 Server, CBS, Technical University of Denmark, Lyngby, Denmark). Several trafficking processes in plants involve post-translational phosphorylation [41]; therefore, a similar mechanism could be involved in the regulation of AtRMR2 localization and trafficking. Additional experiments of mutagenesis would be necessary to identify a trafficking motif within this 23 residues-long cytosolic sequence.

### 3.4. AtRMRs Dimerize in the Secretory Pathway

Our experiments demonstrated that AtRMR1 changed localization from ER to punctate structures when AtRMR2 was co-expressed in the same cell (Figure 7a–c), suggesting interactions between these two receptors that enable the re-localization of AtRMR1 to the TGN. In this case the linker sequence of AtRMR2 is responsible for AtRMR1/AtRMR2 export from the ER and accumulation in this post-Golgi compartment. Furthermore, co-localization experiments between the full length AtRMR2 and two truncated versions of AtRMR1 lacking either the PA domain or the RING-H2 domains along with the short Ser-Rich tail showed that both AtRMR1 mutants changed localization to AtRMR2-labeled punctate structures (Figure 7d–i). Despite the PA and RING-H2 domains of AtRMRs being potential protein interaction domains [42,43], our co-localization experiments suggest that these main protein domains are not necessary for protein–protein interaction.

Compared to the AtRMR1 localization in *Arabidopsis* protoplasts shown by Park et al. [24], the localization we observed in *N. benthamiana* epidermis might be explained by the absence of an RMR2 homolog able to dimerize with AtRMR1 and affect its localization. On the contrary, when the two receptors were overexpressed in the same cells, AtRMR2 was produced in sufficient amounts for AtRMR1 re-localization to the TGN. In *A. thaliana* AtRMR1 and -2 have similar tissue expression profiles with a 30-old excess of AtRMR2 mRNA (as compiled by Genevestigator, NEBION AG, Zurich, Switzerland), and therefore, under normal conditions AtRMR1 probably co-localizes with AtRMR2 in the membrane of TGN, supporting the result of Park et al. [24] obtained in *A. thaliana* protoplasts.

By a BiFC assay we confirmed the AtRMR1–AtRMR2 interaction and also demonstrated the formation of AtRMR2 homodimers (Figure 8b,c,e,f respectively). The homodimerization produced a stronger fluorescence signal than the heterodimerization, suggesting that AtRMR2 homodimers could be more stable then AtRMR1–AtRMR2 heterodimers. On the contrary, no signal was detected for AtRMR1 homodimer formation (Figure 8d), suggesting that it does not occur. The ability of AtRMR1 to leave the ER only after heterodimerization, also suggests that AtRMR1 is retained in the ER because of its inability to homodimerize and that the linker of AtRMR2, rather than containing amino acid motifs directly involved in protein targeting, could be involved in dimer formation.

The aforementioned experiments of BiFC and co-localization using deletion mutants strongly suggest that the main protein domains of AtRMRs (PA, RING-H2 and Ser-Rich domains) are not essential for protein-protein interaction and that the transmembrane domain (TM) and/or the flanking luminal and cytosolic linkers are involved in protein dimerization. This is similar to the finding that both the transmembrane domain and the short cytosolic tail of AtVSR1 (a member of the second family of vacuolar receptors) are also involved in homodimerization [30]. It is well known that membrane proteins can dimerize by interactions between their transmembrane domains [44], and that specific amino acid motifs located in these domains can stabilize helix-helix interactions by forming hydrogen bonds. Several threonine (T) and serine (S) residues present in the TM of AtRMR1 and -2 could constitute such motifs and contribute to their dimerization.

Furthermore, by co-expressing the AtRMR2 pair of BiFC fusion proteins (AtRMR2∆RingSer-nYFP and AtRMR2∆RingSer-cYFP) with AtRMR1∆Ring-RFP, we observed strong AtRMR2∆RingSer split-YFP signal, while the AtRMR1 reporter was not detectably exported from the ER to the TGN (Figure 9). This result further supports a preferential homodimerization of AtRMR2 and the inability to form trimers or tetramers with AtRMR1. However, it is also possible that the association of nYFP and cYFP further stabilized the AtRMR2∆RingSer homodimers and thus excluded even more AtRMR1ΔRing-RFP from contributing to heterodimers.

### 3.5. AtRMR Traffic and Dimerization

AtRMR1 belongs to a separate RMR subfamily, which dates back to the origins of angiosperms, since monocots and even the most basal dicot *Amborella trichopoda* have homologs. On the other hand, several angiosperms seem to have lost this subfamily entirely, including rice, legumes and (more relevant here) *Solanaceae* (Supplementary Material Figure S1). AtRMR1 seems to have lost both the capacity to homodimerize and to leave the ER to traffic to the TGN. It may have complemented these defects by associating (and coevolving) with AtRMR2, the most highly expressed RMR in *Arabidopsis*. In *N. benthamiana*, there is most probably no AtRMR1 homolog and the RMR2 homologs may have diverged enough to be unable to dimerize with AtRMR1.

**Figure 10:**
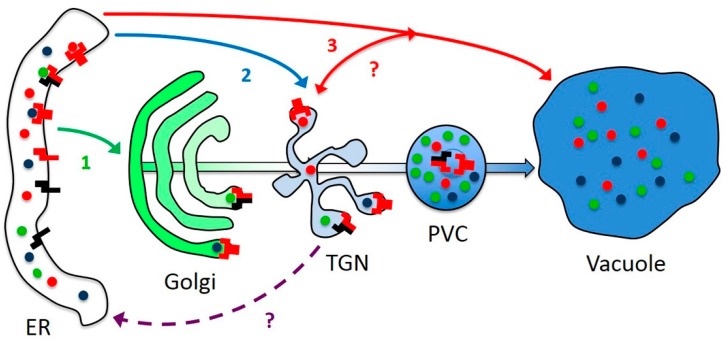
Model of traffic and dimerization of AtRMR1 and -2. AtRMR2 homodimers and AtRMR2/AtRMR1 heterodimers assemble in the ER and can bind specific vacuolar proteins. Homo- and heterodimers could have different specificities for different cargoes (blue, red and green dots). The main pathway from the Golgi to the vacuole is indicated as cisternal maturation without anterograde vesicle transport [2]. Two different pathways could lead AtRMRs to the TGN, either passing through (1, green arrow) or bypassing (2, blue arrow) the Golgi apparatus. A direct ER-to-vacuole sorting mechanism (3, red arrow) is also shown, which would allow the possibility of exchanging material with the TGN (red double-heads arrow). The fate of RMRs after reaching the TGN is unclear. They could either be included into the internal vesicles of the PVC and be degraded in the vacuole (bold-blue arrow from PVC to Vacuole) or they could be recycled back to the ER (dashed arrow). ER, endoplasmic reticulum; Golgi apparatus; TGN, *trans*-Golgi network; PVC, prevacuolar compartment; and vacuole. AtRMR1 and -2 are represented with black and red symbols, respectively.

While its homologs are obviously dispensable in some species, AtRMR1 could still have a specialized function, e.g., AtRMR2 homodimers and AtRMR1/2 heterodimers could have different ligand specificities. Since by itself AtRMR1 remains in the ER, dimerization is likely that occurs there and ligand binding could thus also occur at this early stage of intracellular trafficking. The linker sequence of AtRMR2 is then responsible for AtRMR dimers export from the ER and accumulation in a post-Golgi compartment, identified as TGN. While both dimers accumulate in the same compartment (TGN), they could also be transported by different mechanisms, either trafficking via the Golgi apparatus or bypassing it (Figure 10). Indeed, we did not observe Golgi labeling by our AtRMRs constructs. This could explain why trafficking of the vacuolar reporter GFP-Chi (GFP with the C-terminal VSD of tobacco chitinase A), a probable ligand of RMRs, can occur both ways [45,46,47]. In some plants it might even traffic via PAC vesicles directly from the ER to the PSV, bypassing all intermediate compartments (Figure 10). Whether RMRs form stable dimers or fluctuate between monomers and dimers could not be defined but a member of the other family of vacuolar receptors, AtVSR1, was detected in both monomeric and oligomeric forms [30], suggesting that AtRMRs could be present in both forms. Dimerization could be induced by ligand binding and could initiate ER export.

RMRs may share several aspects of their function with VSRs but may also have peculiarities that need to be elucidated. If the dimerization regulates ER export, it remains to be defined how AtRMR dimers reach the TGN and whether they are recycled back to the ER after transport of vacuolar proteins or incorporated into internal vesicles of the PVC and then degraded in the vacuole (Figure 10). The presence of many arginines (R) in the cytosolic linker of AtRMR2 rather than a typical Tyr or Ile-Met “dileucine” motif, suggests different mechanism of trafficking comparing to AtVSRs. Recently, it has been hypothesized that RMR proteins are not recycled back to Golgi/TGN but incorporated into internal vesicles and then directly sorted to the vacuole [48]. The localization of AtRMR dimers in the TGN described in this work and the absence of vacuolar labeling do not seem to support this hypothesis, therefore further experiments will be needed to decipher the mechanism involved in the trafficking of this family of putative vacuolar receptors.

Furthermore, the trafficking of certain vacuolar proteins to storage vacuoles may occur by a complex ER-to-vacuole pathway in which material exchange with TGN could occur. TGN cisternae, generated independently by hundreds of fully functional dictyosomes [49], are already indicated as a hub where vacuolar targeting, exocytosis and endocytosis merge and their complexity may be still far from being elucidated.

## 4. Materials and Methods

### 4.1. Plant Material and Growth Condition

*Nicotiana benthamiana* plants were grown in a growth chamber Mobylux GroBanks (CLF Plant Climatics, Wertingen, Germany) at a light intensity of 120 μE/m^2^·s with a photoperiod of 16 h of light and 8 h of darkness. The temperature was kept at 22 °C during the day and 20 °C for the night, while the humidity was kept constant at about 70%. *N. benthamiana* plants were grown under non-sterile conditions using soil (RICOTER, Aarberg, Switzerland) containing: 45% sand; 10% perlite; 25% compost; 20% peat.

### 4.2. Agro-Infiltration of N. benthamiana Leaves

*Agrobacterium tumefaciens* strain GV3101 was transformed with plasmids by electroporation and grown at 28 °C overnight under shaking in 5 mL of selective LB (Luria-Bertani) medium-liquid, containing 50 μg/mL kanamycin, 5 μg/mL tetracycline and 50 μg/mL rifampicin. After one day, 1 mL of each pre-culture was inoculated in 50 mL of fresh selective LB medium-liquid and incubated at 28 °C until it reached an OD_600_ of 0.6–1. Bacterial cultures were centrifuged at 5000× *g* for 10 min at room temperature, and then bacterial pellets were washed several times in agro-infiltration buffer containing 50 mM MES, 2 mM Na_3_PO_4_, 0.5% glucose (pH 5.6). After the last wash, bacterial cells were resuspended in agro-infiltration buffer containing 100 μM acetosyringone at an optical density (OD_600_) of about 0.5. The bacterial cells were incubated for 1–2 h at room temperature in the dark and then infiltrated into 4–5 week-old *N. benthamiana* leaves [50]. To increase the protein expression, *A. tumefaciens* cultures transformed with AtRMRs constructs were co-infiltrated with a second *A. tumefaciens* strain carrying a plasmid with a T-DNA encoding the silencing inhibitor p19 [51].

### 4.3. Confocal Microscopy

Infiltrated leaves were imaged 1 day (24 h), 2 days (48 h) and 3 days (72 h) after infiltration with a TCS SP5 II confocal laser scanning microscope (Leica Microsystems, Heerbrugg, Switzerland). The reporters GFP, YFP, CFP and Venus were excited with an argon laser, whereas mRFP and mCherry were excited with a HeNe laser. Digital images were acquired using LAS AF (version: 2.0.0 build 1934, Leica Microsystems, Heerbrugg, Switzerland) and processed using ImageJ 1.41o (National Institute of Health, Bethesda, MD, USA). Single images or stacked images are presented.

### 4.4. Total RNA Extraction and cDNA Synthesis

0.25 g of *A. thaliana* Col-0 leaves were collected in 2 mL RNase free-tube, grinded in liquid nitrogen using a pestle and then 500 μL of plant RNA purification reagent (Thermo Fisher Scientific/Invitrogen, Reinach, Switzerland) were added. The tissue was resuspended, incubated at room temperature for 5 min, and then centrifuged (Eppendorf 5417R benchtop centrifuge, Schönenbuch/Basel, Switzerland) for 2 min at 15,000× *g* at room temperature. One hundred microliters of 5M NaCl were added to the supernatant contained in a new 2 mL RNase free-tube. Three hundred microliters of chloroform were added, and after gently mixing 3–4 times inverting the tube, the sample was centrifuged 10 min at 15,000× *g* at 4 °C. One volume of isopropanol was added to the aqueous phase, and then the total RNA content was precipitated by centrifugation for 30 min at 15,000× *g* at 4 °C. The RNA pellet was washed one time using 75% (*v*/*v*) ethanol and then the pellet was resuspended in an adequate volume of H_2_O RNase free. The contaminant genomic DNA was eliminated by treating the total RNA preparation with DNase (Promega, Dübendorf, Switzerland).

For the total cDNA synthesis, 1 μL of primer oligo-dT and 1 μL of 10 mM dNTP were added in 1.5 mL RNase free-tube containing 1 μg of the aforementioned RNA preparation. The volume of the sample was adjusted to 11 μL using H_2_O RNase free, and then incubated for 5 min at 70 °C and kept for 1 min on ice. Twelve microliters of reaction mix containing 5 μL of 5× transcriptase buffer, 1 μL of 0.1 M DTT, 0.5 μL of SuperScript III RT (Thermo Fisher Scientific/Invitrogen, Reinach, Switzerland) and 5.5 μL of H_2_O RNase free was added to the sample. Thereafter, the reverse transcriptase reaction was performed for 1 h incubating the sample at 50 °C. The reverse transcriptase was inactivated at 70 °C for 15 min and then the sample was transferred on ice. The single strand cDNA preparation was used to amplify *AtRMR* full-length cDNAs using pairs of primers designed on CDS (coding DNA sequence) of *AtRMR1* (TAIR reference: At5g66160; NCBI reference sequence: NM_126014.4) and *AtRMR2* (TAIR reference: At1g71980; NCBI reference sequence: NM_105856.6).

### 4.5. Constructions and Plasmids

The binary Ti vector pGREEN0229 and the associated helper plasmid pSOUP [52] were used for plant transformation via *Agrobacterium tumefaciens*. The cauliflower mosaic virus (CaMV) 35S promoter and terminator from the plant expression vector pGY1 [53] were cloned into *Xho*I/*Sac*I restriction sites located in the multi cloning site (MCS) of pGREEN0229, generating the pGREEN-35S plasmid. This vector was used for the expression in plant cells of both wild-type and mutants AtRMRs. In Supplementary Material Tables S2 and S3, a list of DNA constructs and primers generated in this study are indicated, respectively.

#### 4.5.1. Vectors for C-Terminal Fusion with Different Fluorescent Reporters

A multi cloning site (MCS; *Bam*HI, *Eco*RI, *Xba*I, *Hind*III, *Spe*I and *Nde*I), the sequence coding for a polyglycin spacer (*Gly_6_*) and a HA tag (*HA*) were fused at the 5′ end of the genes encoding the yellow (*YFP*), the cyan (*CFP*) or enhanced green fluorescent protein (*eGFP*) by sequential PCR, using as templates plasmids. Primers 1 and 25 (supplementary material Table S3) were used for the first PCR, followed by a second PCR using primers 4 and 25. The *MCS*:*Gly_6_*:*HA*:*YFP*, *MCS*:*Gly_6_*:*HA*:*CFP* and *MCS*:*Gly_6_*:*HA*:*GFP* fragments (5′-*Bam*HI and 3′-*Sal*I) were then cloned into the *Bam*HI and *Sal*I sites between the 35S promotor and terminator of pGREEN-35S, generating the vectors pGREEN-YFP, pGREEN-CFP and pGREEN-GFP, respectively.

A similar procedure was also used to add a *MCS*, *Gly_6_* and the sequence encoding a Myc tag (*Myc*) to the 5′ end of the gene encoding the monomeric red fluorescent protein (*RFP*). Primers 3 and 27, then 5 and 27, were used to generate the *MCS*:*Gly_6_*:*Myc*:*RFP* fragment (5′-*Bam*HI and 3′-*Sal*I), which was cloned into pGREEN-35S as above, generating the pGREEN-RFP vector.

#### 4.5.2. Vectors for N-Terminal Fusion with Different Fluorescent Reporters

Two sequential PCR using primers 6 and 28, then 7 and 29 were used to amplify the *YFP* gene fused at 5′ end with the sequence encoding the AtRMR2 signal peptide (*Sp2*, aa 1–20) and at 3′ end with the *Gly_6_:Myc*:*MCS* fragment (MCS; *EcoR*I, *Xba*I, *Hind*III, *Spe*I and *Sal*I). The *Sp2*:*YFP*:*Gly_6_*:*Myc*:*MCS* fragment (5′-*Bam*HI and 3′-*Sal*I) was then cloned into pGREEN-35S, generating the pGREEN-Sp2YFP vector.

Similar protocols were used to generate the pGREEN-Sp1YFP and pGREEN-Sp1mCHERRY vectors. The primers 8 and 28, 9 and 29 or 10 and 28, 9 and 29 were used to add the sequence encoding the signal peptide of AtRMR1 (*Sp1*, aa 1–27) at the 5′ end of *YFP* and *mCHERRY*, respectively, and at their 3′ ends the *Gly_6_*:*Myc*:*MCS* fragment. The *Sp1*:*YFP*:*Gly_6_*:*Myc*:*MCS* and *Sp1*:*mCherry*:*Gly_6_*:*Myc*:*MCS* fragments (5′-*Bam*HI and 3′-*Sal*I) were cloned into pGREEN-35S as above, generating pGREEN-Sp1YFP and pGREEN-Sp1mCHERRY, respectively.

#### 4.5.3. Vectors for Bimolecular Fluorescent Complementation (BiFC)

The *YFP* gene was split in two fragments for BiFC as described before [31,32]. Two DNA fragments were generated by PCR, encoding either an N-terminal YFP fragment (*nYFP*, aa 1–155) fused at its 5′ end with *MCS*:*Gly_6_*:*HA* or a C-terminal YFP fragment (*cYFP*, aa 156–239) fused at its 5′ end with *MCS:Gly_6_:Myc*. A 5′-*Bam*HI and 3′-*Sal*I sites were introduced at two ends of both fragments. The primer pairs 1 and 26 and 4 and 26 were used to generate the *nYFP* fragment, while the primer pairs 2 and 25 and 4 and 25 were used to generate the *cYFP* fragment. The *MCS*:*Gly_6_*:*HA*:*nYFP* and *MCS:Gly_6_:Myc:cYFP* fragments were then cloned into pGREEN-35S, generating pGREEN-nYFP and pGREEN-cYFP, respectively.

#### 4.5.4. Constructs for the Expression of Full-Length AtRMR1 and -2

The primer pair 14 and 33 was used to amplify the full-length *AtRMR1* cDNA from a total cDNA preparation from *A. thaliana* Col-0 leaves. This cDNA was cloned into the *EcoR*I and *Hind*III sites of pGREEN-YFP to generate pGREEN AtRMR1-YFP. A cDNA lacking the signal peptide encoding sequence (*AtRMR1ΔSp1*, aa 26–310) was amplified using the primer pair 13 and 32 and cloned into the *EcoR*I and *Hind*III sites of pGREEN-Sp1YFP and pGREEN-Sp1mCHERRY, generating pGREEN YFP-AtRMR1 and pGREEN CHERRY-AtRMR1, respectively.

The primer pair 11 and 30 was used to amplify the full-length *AtRMR2* cDNA from a total cDNA preparation from *A. thaliana* Col-0 leaves. This cDNA was cloned into the *EcoR*I and *Spe*I sites of pGREEN-YFP, pGREEN-GFP or pGREEN-RFP, generating pGREEN AtRMR2-YFP, pGREEN AtRMR2-GFP and pGREEN AtRMR2-RFP, respectively. A cDNA lacking the signal peptide encoding sequence (*AtRMR2ΔSp2*, aa 21–448) was amplified using the primer pair 12 and 31 and cloned into the *EcoR*I and *Spe*I sites of pGREEN-Sp1YFP, generating pGREEN YFP-AtRMR2.

#### 4.5.5. Constructs for the Expression of AtRMR2 and 1 Deletion Mutants

A description of AtRMRs domains used in this study is shown in Supplementary Material Figure S2.

To delete the RING-H2 domain and the short Ser-Rich tail of AtRMR1 (*AtRMR1ΔRing*, aa 1–217), the cDNA was amplified using the primer pair 14 and 38. This fragment was inserted into the *EcoR*I and *Hind*III sites of either pGREEN-YFP or pGREEN-RFP generating pGREEN AtRMR1ΔRing-YFP and pGREEN AtRMR1ΔRing-RFP, respectively. To delete the PA domain, (*AtRMR1ΔPA*, aa 148–310) the cDNA was amplified using the primer pair 16 and 32 and then cloned into the same sites of pGREEN-Sp1YFP, generating pGREEN YFP-AtRMR1ΔPA.

To delete the Ser-Rich domain of AtRMR2 (*AtRMR2ΔSer*, aa 1–279), or its RING-H2 and Ser-Rich domains (*AtRMR2ΔRingSer*, aa 1–207), the cDNA was amplified using the primer pairs 11 and 34 or 11 and 35, respectively. These fragments were then cloned into the same sites of pGREEN-YFP, generating pGREEN AtRMR2ΔSer-YFP and pGREEN AtRMR2ΔRingSer-YFP, respectively. To delete the PA domain (*AtRMR2ΔPA*, aa 145–448), and both the PA and the Ser-Rich domain (*AtRMR2ΔPASer*, aa 145–279), the cDNA was amplified using the primer pairs 15 and 36 or 15 and 37, respectively. These fragments were then cloned into the same sites of pGREEN-Sp2YFP, generating pGREEN YFP-AtRMR2ΔPA and pGREEN YFP-AtRMR2ΔPASer, respectively.

#### 4.5.6. Constructs for the Expression of AtRMR2 and 1 Replacement Mutants of the Transmembrane Domain and Sequence Linker

In order to exchange the trans-membrane of AtRMR2 (*TM2*, aa 162–184) and AtRMR1 (*TM1*, aa 168–190), the sequences encoding the luminal domains extended with part of the other TM, and of the cytosolic domains preceded by the rest of the other TM were amplified. The cDNA sequence encoding the luminal domain of AtRMR2 was amplified with the primer pair 11/39, while the sequence encoding the cytosolic domain by the primer pair 17/30, followed by 18/30. The two fragments were joined at an *Eae*I site within the TM1 encoding sequence and inserted into the *EcoR*I and *Spe*I sites of pGREEN-GFP to generate pGREEN AtRMR2TM1-GFP. Similarly, the cDNA sequence encoding the luminal domain of AtRMR1 was amplified with the primer pair 14/40, followed by 14/41, while the sequence encoding the cytosolic domain by the primer pair 19/44. The two fragments were joined at a *Hind*III within the TM1 encoding sequence and inserted into the *EcoR*I and *Nde*I sites of pGREEN-GFP. The *Hind*III site was then cut, blunt-ended by Mung Bean Nuclease (Promega, Dübendorf, Switzerland) and re-ligated, generating *AtRMR1TM2-GFP*.

The same strategy was used to exchange the linkers of AtRMR1 (*L1*, aa 191–217) and AtRMR2 (*L2*, aa 185–207). The cDNA sequence encoding the luminal and TM domain of AtRMR2TM1 was amplified and extended with a part of the L1 with the primer pairs 11/42 followed by 11/43, while the sequence encoding the cytosolic domain was amplified by the primer pairs 20/30 followed by 21/30. The two fragments were joined at an *EcoR*II site within the L1 encoding sequence and inserted into the *EcoR*I and *Spe*I sites of pGREEN-GFP to generate pGREEN AtRMR2TM1L1-GFP. Similarly, the cDNA sequence encoding the luminal and TM domain of AtRMR1TM2 was amplified and extended with a part of the L2 with the primer pair 14/45, while the sequence encoding the cytosolic domain by the primer pair 22/44. The two fragments were joined at an *Ava*II site within the L2 encoding sequence and inserted into the *EcoR*I and *Nde*I sites of pGREEN-GFP to generate pGREEN AtRMR1TM2L2-GFP.

#### 4.5.7. Constructs for the Expression of Protein Markers of Different Compartments

The cDNA encoding p6 from the Beet Yellow Virus (BYV) was provided by Peremyslov et al. [27] and amplified using the primer pair 23/46. This fragment was cloned as an *EcoR*I/*Spe*I fragment into pGREEN-CFP, generating the pGREEN p6-CFP construct. The cDNA encoding AtGONST1 (*AtGONST1*) [54] was amplified from a total cDNA preparation from *A. thaliana* Col-0 leaves using the primer pair 24/47. This fragment was cloned as an *EcoR*I/*Spe*I fragment into pGREEN-RFP, generating the pGREEN GONST1-RFP. The cDNA fragment *Venus-SYP61* was removed from the pUC18Venus-SYP61 plasmid [28] using *Nco*I/*Sal*I, and then blunt-ended by Mung Bean Nuclease (Promega, Dübendorf, Switzerland) and cloned between the 35S promoter and terminator of pGREEN-35S, generating pGREEN Venus-SYP61.

#### 4.5.8. Constructs for Bimolecular Fluorescent Complementation (BiFC)

The *AtRMR2ΔRingSer* and *AtRMR1ΔRing* sequences (see above) were cloned into *EcoR*I and *Hind*III sites of pGREEN-nYFP and pGREEN-cYFP, generating pGREEN AtRMR2ΔRingSer-nYFP/-cYFP and pGREEN AtRMR1ΔRing-nYFP/-cYFP, respectively. As positive control, the cDNA encoding BYV p6 (see above) was cloned into the *EcoR*I and *Spe*I sites of the pGREEN-nYFP and pGREEN-cYFP vectors, generating pGREEN p6-nYFP and pGREEN p6-cYFP, respectively.

## 5. Conclusions

These experiments provide new information about the mechanisms of the trafficking in the plant secretory pathway of putative vacuolar receptors from the AtRMR family (Receptor Membrane RING-H2) and about their dimerization. In our experiments in agroinfiltrated *N. benthamiana* leaves, one member of each different RMR subfamilies, AtRMR1 and AtRMR2, localized in different subcellular compartment of epidermal cells, the endoplasmic reticulum (ER) and the *trans*-Golgi network (TGN), respectively. By expressing deletion and replacement mutants, we demonstrated that the main RMR protein domains, i.e., PA, transmembrane, RING-H2 or Ser-Rich domains, are not involved in protein localization, while a short sequence linker (L) of ~23-amino acid in the cytosolic part of AtRMR2 is involved in protein localization to the TGN. Further investigations will be necessary to identify trafficking motifs in this cytosolic sequence linker. Moreover, by using a Bimolecular Fluorescent Complementation (BiFC) technique, we demonstrated that AtRMR2 is able to form either homodimers or heterodimers with AtRMR1, which is then able to re-locate to the TGN. These experiments performed using AtRMR deletion mutants also suggest that the transmembrane domain and/or the sequence linker are essential for protein–protein interaction.

## Supplementary Materials

Supplementary materials can be found at www.mdpi.com/1422-0067/17/10/1661/s1.

## Abbreviations

35S	Cauliflower Mosaic Virus (CaMV) promoter
aa	Amino acid
BiFC	Bimolecular Fluorescence Complementation
BP80	Binding Protein of 80 kDa
BYV	Beet Yellow Virus
CFP	Cyan Fluorescent Protein
cYFP	C-terminal YFP fragment
DV	Dense Vesicle
eGFP	Enhanced GFP
ER	Endoplasmic Reticulum
Gly_6_	Spacer composed by 6 Glycines
GFP	Green Fluorescent Protein
GONST1	Golgi localized GDP-mannose Transporter
HA tag	Human Influenza Hemagglutinin (HA) derived protein-tag
L	Cytosolic sequence linker from AtRMRs
LV	Lytic Vacuole
MCS	Multi Cloning Site
Myc	Protein-tag derived from the c-myc gene
mRFP	Monomeric RFP
mCherry	mCherry Fluorescent Protein
nYFP	N-terminal YFP fragment
p6	6 kDa protein from BYV
PA	Protein Associated Domain
PAC	Precursor Accumulating Vesicle
PCR	Polymerase Chain Reaction
PSV	Protein Storage Vacuole
PVC	Prevacuolar Compartment
R	Arginine
RING-H2	RING (Really Interesting New Gene)-H2 domain
RFP	Red Fluorescent Protein
RMR	Receptor-like Membrane RING-H2
S	Serine
ssVSD	Sequence Specific VSD
Ser-Rich	Domain Rich in Serines
Sp	Signal Peptide
SYP61	Syntaxin protein 61
T	Threonine
TGN	*Trans*-Golgi Network
TM	Transmembrane Domain
Venus	Venus Fluorescent Protein
VSD	Vacuolar Sorting Determinant
VSR	Vacuolar Sorting Receptor
YFP	Yellow Fluorescent Protein
