# Supplementary material for: Dimerization of the Vacuolar Receptors AtRMR1 and -2 from Arabidopsis thaliana Contributes to Their Localization in the trans-Golgi Network
Source: Int J Mol Sci. 2016 Sep 30;17(10):1661. doi: 10.3390/ijms17101661 (PMC5085694; doi:10.3390/ijms17101661)
Supplement: Supplementary file 1 [file ijms-17-01661-s001.pdf]

Supplementary Materials: Dimerization of the Vacuolar Receptors AtRMR1 and 2 from *Arabidopsis thaliana* Contributes to Their Localization in the *trans*-Golgi Network

Alessandro Occhialini, Guillaume Gouzerh, Gian-Pietro Di Sansebastiano and Jean-Marc Neuhaus

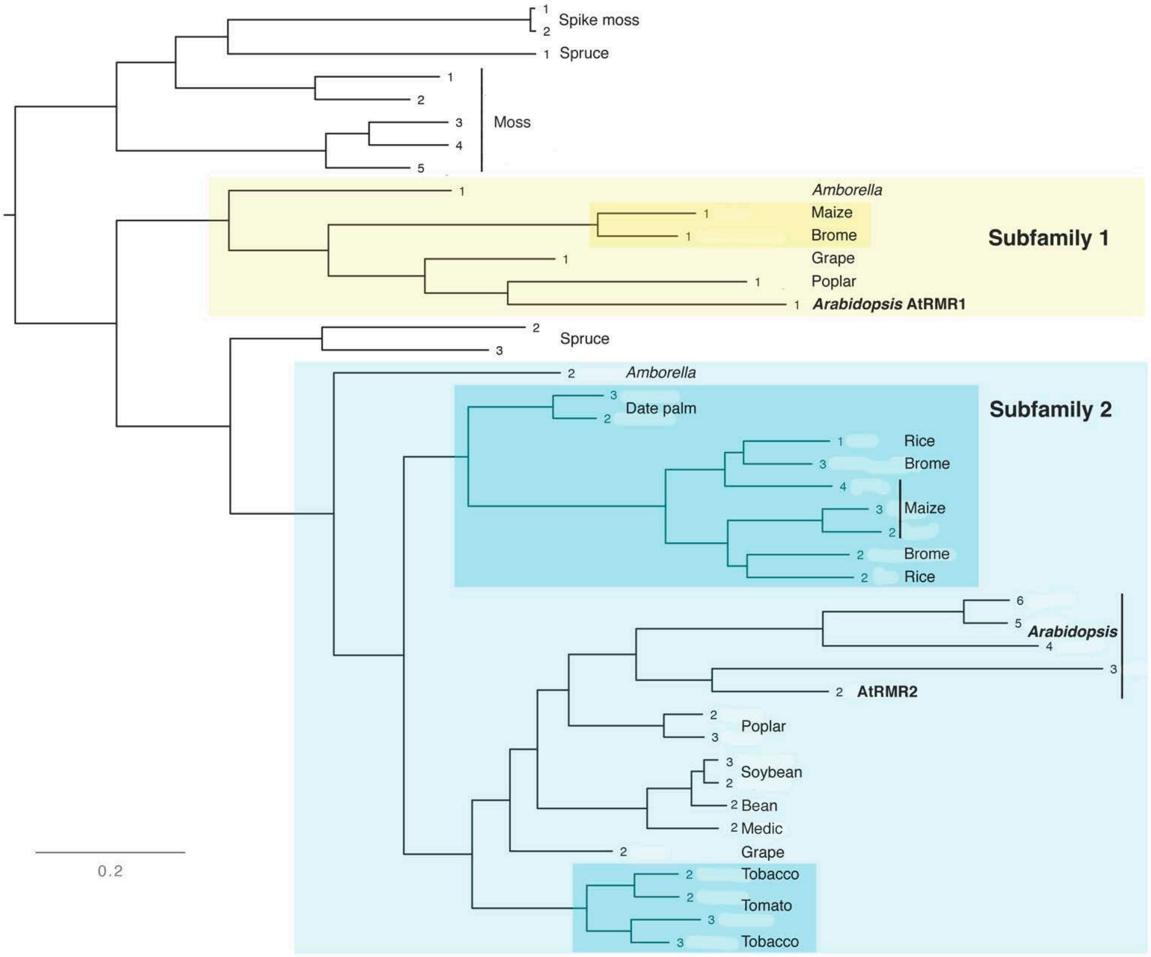

Figure S1. Phylogenic tree of Receptor Membrane RING-H2 (RMRs). RMR coding sequences from representative species were aligned using ClustalΩ (EMBL-EBI). The phylogeny was then calculated with the DNAML (Maximum likelihood) program from the PHYLIP package [1], using human RNF13 as root. The numbers at the end of the branches are the RMR numbers for the RMR sequences from each species as indicated in Supplementary Table S1. For angiosperms RMR sequences from subfamily 1 were numbered RMR1, while RMR sequences from subfamily 2 were numbered RMR2, 3, etc., with the exception of the already published rice RMR1 and 2. Gymnosperms also have two subfamilies but the relationship to the angiosperm subfamilies is unclear. In angiosperm RMR subfamilies 1 (yellow) and 2 (blue) the monocots are highlighted. In subfamily 2 the *Solanaceae* are also highlighted. They are absent in subfamily 1, as are also rice and legumes. Species included: the spikemoss *Selaginella moellendorffii* (lycopodiophyte), the moss *Physcomitrella patens* (bryophyte), the spruce *Picea sitchensis* (gymnosperm), the basal angiosperm *Amborella trichopoda* ; the monocots maize (*Zea mays*), rice (*Oryza sativa*), stiff brome (*Brachypodium distachyon*) and date palm (*Phoenix dactylifera*); the eudicots *Arabidopsis thaliana*, poplar (*Populus trichocarpa*), soybean (*Glycine max*), bean (*Phaseolus vulgaris*), medic (*Medicago truncatula*), grapevine (*Vitis vinifera*), tobacco (*Nicotiana sylvestris*) and tomato (*Solanum lycopersicum*).

```
                Sp1                                    PA1
AtRMR1  MRLVVSSCLLVAAPFLSSLLRVSLATVVLNSISASFADLPAKFDGSVTKNGICGALYVAD
AtRMR2  MNRALVLLLYVCT---VSCLASSKVILMRNNITLSFDDIEANFAPSVKGTGEIGVVYVAE
                Sp2                                    PA2

AtRMR1  PLDGCSPLLH--AAASNWTQHRTTKFALIIRGECSFEDKLLNAQNSGFQAVIVYDNIDNE
AtRMR2  PLDACQNLMNKPEQSSNETSP----FVLIVRGGCSFEEKVRKAQRAGFKAAIIYDNEDRG

                  PA1                                           TM1
AtRMR1  DLIVMKVNPQDITVDAVFVSNVAGEILRKYARGRDGECCLNPPDRGSAWTVLAISFFSLL
AtRMR2  TLIAMAGNSGGIRIHAVFVTKETGEVLKEYAGFPDTKVWLIPSFENSAWSIMAVSFISLL
                  PA2                                           TM2

             TM1               L1                             Ring1
AtRMR1  LIVTFLLIAFFAPRHWTQWRGRHTRTIRL------DAKLVHTLPCFTFTDSAHHKAG--E
AtRMR2  AMSAVLATCFFVRRHR--IRRRTSRSSRVREFHGMSRRLVKAMPSLIFS-SFHEDNTTAF
             TM2               L2                             Ring2

                  Ring1                                         Ser1
AtRMR1  TCAICLEDYRFGESLRLLPCQHAFHLNCIDSWLTKWGTSCPVCKHDIRTETMSSEVHKRE
AtRMR2
        TCAICLEDYTVGDKLRLLPCCHKFHAACVDSWLTSWRTFCPVCKRDARTSTGEPPASES
T
                  Ring2                                         Ser2

                  Ser1
AtRMR1  SPRTDTSTSRFAFAQSSQSR*
AtRMR2  PLLSSAASSFTSSSLHSSVRSSALLIGPSLGSLPTSISFS-PAYASSSYIRQSFQSSSNR
                  Ser2

AtRMR2  RSPPISVSRSSVDLRQQAASPSPSPSQRSYISHMASPQSLGYPTISPFNTRYMSPYRPSP

AtRMR2  SNASPAMAGSSNYPLNPLRYSESAGTFSPYASANSLPDC*
```

Figure S2. Definition of AtRMR domains as used in this study. The domain limits of protein domains are indicated in the amino acid sequences of AtRMR1 and 2, respectively. The signal peptides were defined by the program SignalP 4.1 Server, while the transmembrane domains were defined by the program TMHMM Server v. 2.0. Signal peptide (Sp, black); PA domain (PA, red); transmembrane (TM, green); linker (L, black); RING-H2 domain (Ring, red); and Ser-Rich domain (Ser, black). (*) C-terminus of amino acid sequence.

Table S1. List of sequences used for the phylogenetic analysis (Supplementary Figure S1). Sequences were obtained from Genbank and are mostly derived from genomic sequences. *Picea sitchensis* are cDNA sequences only. *Selaginella moellendorffii* sequences are derived from unannotated genomic scaffold. *Physcomitrella patens* sequences were obtained from Cosmoss.org. (#) RMR number as explained in legend of Figure S1.

Species	RMR #	Accession Number	Gene ID
Arabidopsis thaliana	RMR1	AK318938	AT5G66160
	RMR2	AY133843	AT1G71980
	RMR3	NM_102114	AT1G22670
	RMR4	NM_117024	AT4G09560
	RMR5	NM_103264	AT1G35630
Amborella trichopoda	RMR1	XM_006846479	LOC18436459
	RMR2	XM_006830360	LOC18425826
Brachypodium distachion	RMR1	XM_003581574	LOC100845565
	RMR2	XM_014901413	LOC100826153
	RMR3	XM_010242369	LOC100844532
Glycine max	RMR2	XM_003556143	LOC100793048
	RMR3	XM_003536341	LOC100791248
Medicago truncatula	RMR2	XM_013614083	MTR_1g096780
Nicotiana sylvestris	RMR2	XM_009770080	LOC104219391
	RMR3	XM_009795991	LOC104241077
Oryza sativa	RMR1	XM_015776897	LOC4331745
	RMR2	XM_015795126	LOC4344437
Phaseolus vulgaris	RMR2	XM_007143576	PHAVU_007G088600g
Phoenix dactylifera	RMR2	XR_604743	LOC103711273
	RMR3	XM_008785170	LOC103702657
Physcomitrella patens	RMR1	Pp1s28_303V6.1	Phypa_429164
	RMR2	Pp1s58_112V6.2	Phypa_435212
	RMR3	Pp1s81_169V6.1	Phypa_438832
	RMR4	Pp1s88_138V6.1	Phypa_439901
	RMR5	Pp1s282_31V6.1	Phypa_455658
Picea sitchensis	RMR1	EF086419	-
	RMR2	EF677639	-
	RMR3	EF082884	-
Populus trichocarpa	RMR1	XM_006383013	POPTR_0005s11320g
	RMR2	XM_002325950	POPTR_0019s11200g
	RMR3	XM_006376215	POPTR_0013s11590g
Selaginella moellendorffii	RMR1	ADFJ01000268	SELMOscaffold_2_Cont268
	RMR2	ADFJ01003771	SELMOscaffold_141_Cont3771
Solanum lycopersicum	RMR2	XM_004241781	LOC101252295
	RMR3	XM_004250357	LOC101248068
Vitis vinifera	RMR1	XM_002265634	LOC100245428
	RMR2	XM_002269695	LOC100246359
Zea mays	RMR1	NM_001150087	LOC100276255
	RMR2	XM_008649494	LOC100279400
	RMR3	XM_008679523	LOC100502318
	RMR4	XM_008676740	LOC100281566

Table S2. The plant expression vectors encoding AtRMRs fusion proteins and different protein markers generated in this study. Vectors encoding: (a) AtRMR1 full-length and deletion mutants; (b) AtRMR2 full-length and deletion mutants; (c) AtRMR replacement mutants of transmembrane and/or linker domains; (d) AtRMRs and p6 fusion proteins for BiFC; (e) protein markers of different subcellular compartments. The binary Ti plasmid was always pGREEN0229 and the constructs were under the control of the 35S promotor.

Construct Group	Construct Name	Encoded Protein
a	pGREEN AtRMR1-YFP	AtRMR1:Spacer:HA:YFP
	pGREEN AtRMR1ΔRing-YFP	AtRMR1ΔRing:Spacer:HA:YFP
	pGREEN AtRMR1ΔRing-RFP	AtRMR1ΔRing:Spacer:Myc:RFP
	pGREEN YFP-AtRMR1	Sp1YFP:Spacer:Myc:AtRMR1ΔSp1
	pGREEN Cherry-AtRMR1	Sp1Cherry:Spacer:Myc:AtRMR1ΔSp1
	pGREEN YFP-AtRMR1ΔPA	Sp1YFP:Spacer:Myc:AtRMR1ΔPA
b	pGREEN AtRMR2-YFP	AtRMR2:Spacer:HA:YFP
	pGREEN AtRMR2-GFP	AtRMR2:Spacer:HA:GFP
	pGREEN AtRMR2-RFP	AtRMR2:Spacer:Myc:RFP
	pGREEN AtRMR2ΔSer-YFP	AtRMR2ΔSer:Spacer:HA:YFP
	pGREEN AtRMR2ΔRingSer-YFP	AtRMR2ΔRingSer:Spacer:HA:YFP
	pGREEN YFP-AtRMR2	Sp2YFP:Spacer:Myc:AtRMR2ΔSp2
	pGREEN YFP-AtRMR2ΔPA	Sp2YFP:Spacer:Myc:AtRMR2ΔPA
	pGREEN YFP-AtRMR2ΔPASer	Sp2YFP:Spacer:Myc:AtRMR2ΔPASer
c	pGREEN AtRMR1TM2-GFP	AtRMR1TM2:Spacer:HA:GFP
	pGREEN AtRMR1TM2L2-GFP	AtRMR1TM2L2:Spacer:HA:GFP
	pGREEN AtRMR2TM1-GFP	AtRMR2TM1:Spacer:HA:GFP
	pGREEN AtRMR2TM1L1-GFP	AtRMR2TM1L1:Spacer:HA:GFP
d	pGREEN AtRMR1ΔRing-nYFP	AtRMR1ΔRing:Spacer:HA:nYFP
	pGREEN AtRMR1ΔRing-cYFP	AtRMR1ΔRing:Spacer:Myc:cYFP
	pGREEN AtRMR2ΔRingSer-nYFP	AtRMR2ΔRingSer:Spacer:HA:nYFP
	pGREEN AtRMR2ΔRingSer-cYFP	AtRMR2ΔRingSer:Spacer:Myc:cYFP
	pGREEN p6-nYFP	p6:Spacer:HA:nYFP
	pGREEN p6-cYFP	p6:Spacer:Myc:cYFP
e	pGREEN p6-CFP	p6:Spacer:HA:CFP
	pGREEN GONST1-RFP	AtGONST1:Spacer:Myc:RFP
	pGREEN Venus-SYP61	Venus:SYP61

Table S3. The oligonucleotides used in the construction of plant expression vectors generated in this study. The oligonucleotide sequences are numbered from 1 to 24 (forward primers) and 25 to 47 (reverse primers).

Primer Number	Primer Name	5′–3′ Sequence
1	Spacer-HA-YFP-n-fw	GGAGGAGGAGGAGGAGGATACCCATACGACGTCCCAGACTACGCTGTGAGCAAGGGCGAGGAGCTG
2	Spacer-Myc-YFP-c-fw	GGAGGAGGAGGAGGAGGAGAACAACAAAAACTTATTTCTGAAGAAGATCTGGACAAGCAGAAGAACGGCATC
3	Spacer-Myc-RFP-fw	GGAGGAGGAGGAGGAGGAGAACAACAAAAACTTATTTCTGAAGAAGATCTGGCCTCCTCCGAGGACGTCATCA
4	MCS-Spacer-fw	GCGCGCGGATCCGAATTCTCTAGAAAGCTTACTAGTCATATGGGAGGAGGAGGAGGAGGA
5	Spacer-fw	GGCCGGGGATCCGAATTCTCTAGAAAGCTTACTAGTCATATGGGAGGAGGAGGAGGAGGAGAACAAC
6	Prim1-Sp2YFP-fw	CTGTTTCTTGTTTAGCTTCAAGCGTGAGCAAGGGCGAGGAGCTGT
7	Prim2-Sp2YFP-fw	GGCCGGGGATCCATGAATCGTGCTTTGGTCCTACTTTTATATGTTTGTACTGTTTCTTGTTTAGCTTCAAG
8	Prim1-Sp1YFP-fw	GCTCCTTTTCTCTCCTCTCTGTTACGAGTCTCACTCGCCACTGTTGTGAGCAAGGGCGAGGAGCTGT
9	Prim2-Sp1YFP-fw	GGCCGGGGATCCATGAGACTCGTCGTCTCAAGCTGTCTACTAGTTGCAGCTCCTTTTCTCTCCTCTCTGT
10	Prim1-Sp1mCHERRY-fw	GCTCCTTTTCTCTCCTCTCTGTTACGAGTCTCACTCGCCACTGTTGTGAGCAAGGGCGAGGAGGATAA
11	EcoRI-RMR2-fw	GCGCGCGAATTCATGAATCGTGCTTTGGTCC
12	EcoRI-delRMR2-fw	GGCCGGGAATTCAAAGTTATTTTGATGAGGAATAACATCA
13	EcoRI-delRMR1-fw	CCGGCCGAATTCACTGTTGTCCTCAATTCCATCT
14	EcoRI-RMR1-fw	CCGGCCGAATTCATGAGACTCGTCGTCTCAAG
15	EcoRI-DP-RMR2-fw	GCGCGCGAATTCGGTTTCCCCGATACGAAAGT
16	EcoRI-delPA-RMR1-fw	CCGGCCGAATTCAGAGGCCGAGATGGTGAATGC
17	RMR2-TM1-fw	ATAGTCACTTTCCTGTTGATTGCCTTCTTTGCAGTGCGTAGGCATCGAATAAGAAGGC
18	RMR2-TM1b-fw	GGCCGGTGGCCATCTCCTTCTTCTCTCTCCTTCTTATAGTCACTTTCCTGTTGATTGCC
19	RMR1-TM2b-fw2	GCGCGCAAGCTTGTCGGCTGTTCTCGCTACTTGTTTCTTTCCCAGACACTGGACCCAATGGCGAG
20	TM1-link1-fw1	CAAAGCTCGTCCACACACTCCCCTGCCGCCGCTTGGTGAAAGCAATGCC
21	TM1-link1-fw2	GGCCGGCCAGGACCATCAGGTTAGATGCAAAGCTCGTCCACACACTCCCC
22	TM2-link2-fw	GCGCGCGGTCCTCTCGAGTGCGTGAGTTTCACGGTATGAGCTTCACCTTCACTGATTCTGCTCACC
23	EcoRI-p6-fw	GCGCGCGAATTCATGGACTGTGTACTCCGCTC
24	EcoRI-GONST1-fw	GGCCGGGAATTCATGAAATTGTACGAACACGATG
25	c-YFP-rev	GCGCGCGTCGACTTACTTGTACAGCTCGTC
26	n-YFP-rev	GCGCGCGTCGACTTAGGCCATGATATAGAC
27	RFP-rev	CCGGCCGTCGACTTATGCTCCAGTACTGTGGCGGCCC
28	Prim1-Spacer-Myc-rev	TCTTCAGAAATAAGTTTTTGTTGTTCTCCTCCTCCTCCTCCCTTGTACAGCTCGTCCATGCCG
29	Prim2-Spacer-Myc-rev	CCGGCCGTCGACACTAGTAAGCTTTCTAGAGAATTCCAGATCTTCTTCAGAAATAAGTTTTTGTTG
30	RMR2-SpeI-rev	GCGCGCACTAGTACAGTCTGGAAGCGAGTTTGC

Table S3. *Cont.*

Primer Number	Primer Name	5′–3′ Sequence
31	delRMR2-SpeI-rev	CCGGCCACTAGTCTAACAGTCTGGAAGCGAG
32	delRMR1-HindIII-rev	CCGGCCAAGCTTCTAACGGCTTTGACTGGATTG
33	RMR1-HindIII-rev	CCGGCCAAGCTTACGGCTTTGACTGGATTGGG
34	DS-RMR2-HindIII-rev	CCGGCCAAGCTTCGTTCTTGCATCTCGTTTGC
35	DRS-RMR2-HindIII-rev	GGCCGGAAGCTTGCTCATACCGTGAAACTCA
36	delRMR2-HindIII-rev	CCGGCCAAGCTTCTAACAGTCTGGAAGCGAG
37	DSP-RMR2-HindIII-rev	CCGGCCAAGCTTCTACGTTCTTGCATCTCGTT
38	delRing-RMR1-HindIII-rev	CCGGCCAAGCTTGCAGGGGAGTGTGTGGACGA
39	RMR2-TM1a-rev	CCGGCCTGGCCAACACAGTCGCCGAGTTCTCAAAACTTGG
40	RMR1-TM2a-A-rev	TAAACGAAACCGCCATAATAGACCACCAAGCGCTCCCTCTGTCTGGCGG
41	RMR1-TM2a-B-rev	CGCGCGAAGCTTTGCAAGCAGCGAGATAAACGAAACCGCCATAATAGACC
42	TM1-link1-rev1	GCCATTGGGTCCAGTGTCTGGGTGCAAAGAAGGCAATCAACAGG
43	TM1-link1-rev2	GGCCGGCCTGGTGTGCCTCCCTCGCCATTGGGTCCAGTGTCTGGGT
44	RMR1-NdeI-rev	CGCGCGCATATGACGGCTTTGACTGGATTGGG
45	TM2-link2-rev	GCGCGCGGACCGAGATGTCCGCCTTCTTATTCGATGCCTACGCACAAAGAAACAAGTAGCGAGAACAGCC
46	p6-SpeI-rev	GCGCGCACTAGTCACGACCGTGGAACGGTTGA
47	GONST1-SpeI-rev	GGCCGGACTAGTGGACTTCTCCCTCATTTTGG

References

1. Felsenstein, J. Phylogenies from molecular sequences: Inference and reliability. *Annu. Rev. Genet.* **1988**, *22*, 521–565.
